# The Effects of Safinamide Adjunct Therapy on Depression and Apathy in Patients With Parkinson's Disease: *Post-hoc* Analysis of a Japanese Phase 2/3 Study

**DOI:** 10.3389/fneur.2021.752632

**Published:** 2022-02-07

**Authors:** Nobutaka Hattori, Yuki Kogo, Michinori Koebis, Takayuki Ishida, Ippei Suzuki, Yoshio Tsuboi, Masahiro Nomoto

**Affiliations:** ^1^Department of Neurology, Juntendo University School of Medicine, Tokyo, Japan; ^2^Medical Headquarters, Eisai Co., Ltd., Tokyo, Japan; ^3^Medicine Development Center, Eisai Co., Ltd., Tokyo, Japan; ^4^Department of Neurology, Fukuoka University, Fukuoka, Japan; ^5^Department of Neurology, Saiseikai Imabari Center for Health and Welfare, Ehime, Japan

**Keywords:** clinical trial, MAO-B inhibitor, depression, apathy, Parkinson's disease, *post-hoc* analysis, safinamide

## Abstract

**Background and Purpose:**

Neuropsychiatric symptoms in Parkinson's disease (PD) have been shown to significantly affect quality of life (QOL). We investigated the impact of safinamide on depression and apathy when administered as an adjunct to levodopa in Japanese patients with PD.

**Methods:**

This was a *post-hoc* analysis of data from a phase 2/3 clinical study of safinamide in Japanese patients with PD experiencing wearing-off (JapicCTI-153056; https://www.clinicaltrials.jp/cti-user/trial/ShowDirect.jsp?japicId=JapicCTI-153056). Patients received placebo, safinamide 50 mg, or safinamide 100 mg as an adjunct therapy. The endpoints for this analysis were changes from baseline to Week 24 in the Unified Parkinson's Disease Rating Scale (UPDRS) Part I item 3 (depression) and item 4 (apathy) scores and the Parkinson's Disease Questionnaire (PDQ-39) “emotional well-being” domain score. Subgroup analyses investigated the relationship between neuropsychologic symptoms and improvements in motor fluctuation and assessed which patient populations might be expected to obtain neuropsychologic benefit from safinamide.

**Results:**

Compared with placebo, safinamide (both doses) significantly improved UPDRS Part I item 3 scores in the overall analysis population, and the 100-mg dose improved UPDRS Part I item 4 scores in the population with apathy at baseline. Changes in the PDQ-39 “emotional well-being” score showed numerical, but not significant, dose-related improvements. Notable reductions in depression were associated with a change in daily ON-time ≥1 h, pain during OFF-time at baseline, and female sex.

**Conclusions:**

The results from this *post-hoc* analysis of the Japanese phase 2/3 study suggest that safinamide could bring benefits to patients with PD who have mild depression, pain during the OFF phase. In addition, safinamide might provide particular benefits for patients with PD who have mild apathy and female.

## Introduction

According to the most recent estimates, Parkinson's disease (PD) affects more than 6 million people worldwide, and the incidence is expected to double within a generation (1). In addition to the well-characterized motor symptoms (2), patients with PD generally experience various non-motor symptoms, of which neuropsychiatric symptoms are a major clinical concern (3, 4). Neuropsychiatric symptoms in PD can include depression (5), anxiety (6), and apathy (7), and they are known to increase in prevalence (8) and worsen with the severity of PD (9). These symptoms have been shown to significantly affect the quality of life (QOL) of patients with PD (10). In a recent survey undertaken in a large population of patients with PD in Japan, a strong correlation was found between mood and QOL (11); thus, improving mood is considered to be an important treatment goal for Japanese patients with PD.

Depression is experienced by approximately 35% of patients with PD (12, 13) and appears to negatively impact QOL (14). In addition to the general risk factors for developing depression in the overall population (15), specific risk factors for depression in PD have been reported. These include being female, having cognitive impairment, and having episodes of psychiatric disease, anxiety, and sleep disorders (16). Depression in PD is related to the severity of motor symptoms, ON/OFF fluctuations, and motor complications (15). However, depression in PD may also be related to other non-motor symptoms. For example, one study has shown an interrelationship between depression and pain in PD, with patients with depression reporting higher pain scores than patients without depression (17), while another study has indicated a link between non-motor symptoms (including depression) and subjective sleep quality (18).

Apathy is another problematic neuropsychiatric symptom in PD. Apathy is defined as a loss of motivation to act toward a goal and decreased interest and emotion (19), that is a series of concurrent behavioral, affective, and cognitive features (20). The prevalence of apathy in patients with PD is approximately 40% (21). Factors associated with apathy in PD include higher age, comorbid depression, exacerbation of motor symptoms, and impairment in activities of daily life (21).

Many patients with advanced PD experience motor fluctuations as a complication of dopamine replacement therapy; however, fluctuations in non-motor symptoms, including psychiatric symptoms, are also common following treatment (22). The observation that symptoms can be exacerbated during the OFF phase (23–25) indicates the dopaminergic system's involvement in neuropsychiatric symptoms in patients with PD. However, dopamine agonists do not always improve non-motor symptoms, such as depression or apathy, and non-dopaminergic approaches are also required (26). Studies have suggested that other neurotransmitters, such as serotonin and norepinephrine, are associated with non-motor symptoms (27). As such, agents targeting the enzymes that metabolize these neurotransmitters are of great interest for treating depression in PD (28).

Safinamide, an antiparkinsonian drug licensed as an add-on therapy for patients with PD who are experiencing motor fluctuations with levodopa (29, 30), has dual mechanisms of action. In addition to being a reversible monoamine oxidase B (MAO-B) inhibitor, safinamide inhibits glutamate release via its interaction with voltage-gated sodium-channels (31). Owing to these two actions, safinamide is hypothesized to contribute to an improvement in non-motor symptoms as well as motor symptoms in patients with PD. In a *post-hoc* analysis of two global phase 3 studies, safinamide was reported to provide significant improvements in the “emotional well-being” domain of the Parkinson's Disease Questionnaire (PDQ-39) and the scores of the GRID Hamilton Rating Scale for Depression (GRID-HAMD) (32).

We conducted a *post-hoc* analysis of a placebo-controlled phase 2/3 study in Japanese patients (33) to investigate the impact of safinamide 50 and 100 mg on neuropsychiatric symptoms, focusing on depression and apathy. In addition, subgroup analyses were performed to investigate the relationship between improvement of neuropsychiatric symptoms and reduction of motor symptoms and determine which patient populations may obtain neuropsychologic benefit from safinamide.

## Materials and Methods

### Study Design and Study Population

Full details of the study design and patient eligibility criteria for the 24-week, randomized, double-blind, placebo-controlled, parallel-group study have been published (33). The study was registered with the identifier JapicCTI-153056 and was conducted in accordance with the International Conference on Harmonization Good Clinical Practice guidelines and the Declaration of Helsinki. All patients (or their legal representatives) provided written informed consent prior to initiation of study procedures. The protocol and its amendments were approved by all appropriate independent ethics committees and the Japanese regulatory authority.

Patients diagnosed with PD who experienced wearing-off phenomena were enrolled at 71 sites in Japan. Additional inclusion criteria were a modified Hoehn & Yahr (H&Y) stage of II–IV during an “OFF” phase and were on levodopa treatment for at least 24 weeks before the study began. Regarding depression, patients who had a history of psychosis including psychotic depression were excluded and use of antidepressants was prohibited during the study. Patients were randomly assigned (1:1:1 ratio) to three treatment groups to receive once daily (morning) doses of safinamide 50 mg, safinamide 100 mg, or placebo, for 24 weeks. All study treatments were administered as an add-on therapy to a stable dose of levodopa.

### Outcome Evaluations

The endpoints assessed in this *post-hoc* analysis were changes from baseline to Week 24 in the Unified Parkinson's Disease Rating Scale (UPDRS) Part I item 3 (depression) and item 4 (apathy) scores, and in the PDQ-39 emotional well-being domain score.

Subgroup analyses of UPDRS Part I item 3 and item 4 scores were conducted in patients categorized according to ON-time response (high responders were defined as patients with a change from baseline in daily ON-time without troublesome dyskinesia ≥1 h, and low/non-responders were defined as patients with a change <1 h), depression at baseline (present or absent), apathy at baseline (present or absent), sex (male or female), use of concomitant dopamine agonist (yes or no), and pain during the OFF phase at baseline (present or absent). The degree of change in ON-time was explored as a factor based on the possibility of improved neuropsychiatric symptoms resulting from improvements in daily motor fluctuations (15). Depression at baseline was defined by a score of >0 on UPDRS Part I item 3. Apathy at baseline was defined by a score of >0 on UPDRS Part I item 4. Pain during the OFF phase at baseline was defined by a score of >0 on UPDRS Part II item 17 (OFF).

### Statistical Methods

Data from patients included in the full analysis set (FAS) of the primary analysis (33) (i.e., patients who had received at least one dose of the study drug and whose ON-time was assessable at baseline and the final evaluation) were used for these *post-hoc* analyses.

The last observation carried forward (LOCF) methodology was used to impute dropout and missing data at the last assessment point. Patient demographic characteristics and baseline values for each endpoint were compared between the population with a baseline UPDRS Part I item 3 score of 0 and the population with a score >0. Welch's *t*-test was used for continuous variables and Fisher's exact test was used for categorical variables. An analysis of covariance (ANCOVA) for comparison of the changes from baseline to the last assessment in the efficacy endpoints between treatment groups was performed with the change from baseline to the last assessment as a response variable, the treatment groups as fixed effects, and the baseline value as a covariate. Investigation of the difference in dose-dependency between subgroups was performed using ANCOVA. Statistical comparisons between the placebo group and each safinamide dose group were performed. All tests had a two-tailed significance level of 5%, and no adjustments were made for multiplicity. All analyses were conducted using SAS version 9.4 (SAS Institute Inc., Cary, NC, USA).

## Results

### Patients

The FAS comprised 131 patients who received safinamide 50 mg, 128 who received safinamide 100 mg, and 136 who received placebo. Demographic data are shown in Supplementary Table 1.

### Impact of Safinamide on Depression and Apathy

Table 1 shows the changes from baseline to Week 24 in scores related to neuropsychiatric symptoms. The changes [least squares mean (LS mean) ± standard error (SE)] in the UPDRS Part I item 3 (depression) scores from baseline to Week 24 were 0.07 ± 0.04, −0.06 ± 0.04, and −0.09 ± 0.04 for the placebo, safinamide 50- and 100-mg groups, respectively. Improvements from baseline in both the safinamide 50- and 100-mg groups were statistically significant (*p* = 0.0095 and *p* = 0.0024, respectively).

**Table 1:** Baseline values and change from baseline to Week 24 in the UPDRS Part I item 3 and item 4 scores, and in the PDQ-39 emotional well-being score.

	**UPDRS part I item 3**			**UPDRS part I item 4**			**PDQ-39 emotional well-being**		
	**Placebo**	**Safinamide**	**Safinamide**	**Placebo**	**Safinamide**	**Safinamide**	**Placebo**	**Safinamide**	**Safinamide**
		**50 mg**	**100 mg**		**50 mg**	**100 mg**		**50 mg**	**100 mg**
*N*	136	131	128	136	131	128	136	131	128
Baseline, mean ± SD	0.31 ± 0.55	0.29 ± 0.56	0.33 ± 0.68	0.39 ± 0.66	0.40 ± 0.70	0.40 ± 0.66	23.28 ± 17.69	29.26 ± 21.27	29.20 ± 22.35
Change from baseline at Week 24 (LOCF), LS mean ± SE	0.07 ± 0.04	−0.06 ± 0.04	−0.09 ± 0.04	0.06 ± 0.04	−0.01 ± 0.04	−0.05 ± 0.04	−1.49 ± 1.22	−2.61 ± 1.24	−3.20 ± 1.26
LS mean difference vs. placebo [95% CI]	—	−0.13 [−0.24, −0.03]	−0.16 [−0.26, −0.06]	—	−0.06 [−0.18, 0.05]	−0.10 [−0.22, 0.01]	—	−1.12 [−4.55, 2.32]	−1.70 [−5.16, 1.76]
*p*-Value vs. placebo	—	0.0095	0.0024	—	0.2692	0.0780	—	0.5242	0.3339

*CI, confidence intervals; LOCF, last observation carried forward; LS, least squares; SD, standard deviation; SE, standard error*.

UPDRS Part I item 4 (apathy) scores also showed dose-dependent improvements (LS mean ± SE changes were 0.06 ± 0.04 for placebo, −0.01 ± 0.04 for safinamide 50 mg, and −0.05 ± 0.04 for safinamide 100 mg), but the differences between the safinamide and placebo groups were not statistically significant (Table 1).

The PDQ-39 emotional well-being score decreased numerically with both doses of safinamide. However, no statistically significant differences from placebo were observed (LS mean ± SE change for the 50-mg dose: −1.12 ± 1.242; *p* = 0.5242; and for the 100-mg dose: −1.70 ± 1.26; *p* = 0.3339) (Table 1).

### Efficacy of Safinamide on Improving Depression (Subgroup Analyses)

To identify the characteristics of the patients with depression, we compared epidemiological variables and baseline characteristics of patients in populations with UPDRS Part I item 3 scores of 0 and > 0 at baseline (Supplementary Table 2). Statistically significant differences in sex (*p* = 0.0049), duration of disease (*p* = 0.0242), OFF-time duration (*p* = 0.0007), UPDRS Part II scores (activities of daily living) (*p* < 0.0001), Part III scores (motor symptoms) (*p* = 0.0006), PDQ-39 summary index (*p* < 0.0001), and emotional well-being domain scores (*p* < 0.0001) were observed between patients with or without depression at baseline. There was also a notable difference in the UPDRS Part II item 17 (pain) score during OFF-time (*p* = 0.0527).

**Figure 1:**
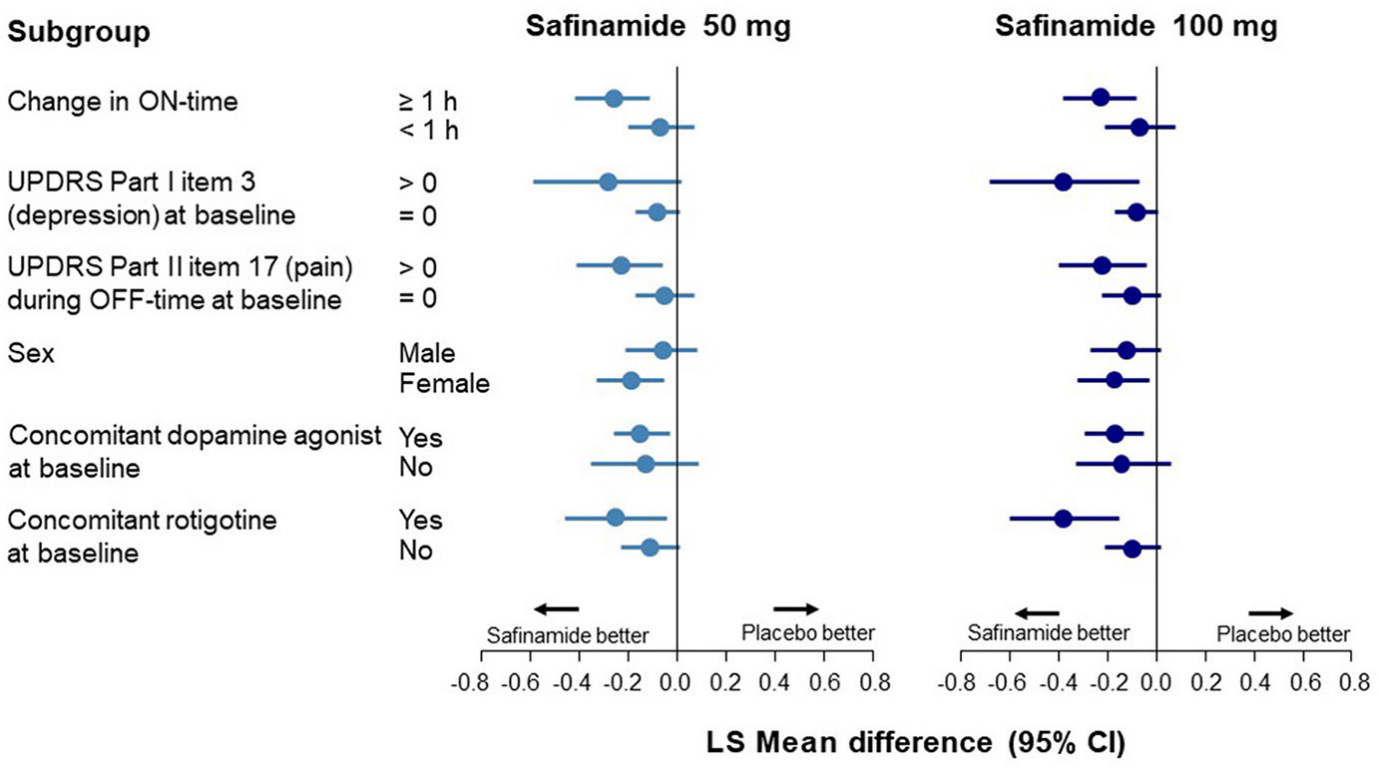
Forest plot of change from baseline to Week 24 in the UPDRS Part I item 3 score according to subgroup. CI, confidence interval; UPDRS, Unified Parkinson's Disease Rating Scale.

Data corresponding to changes from baseline to Week 24 in UPDRS Part I item 3 scores are shown in Figure 1 and Supplementary Table 3. In the ON-time high responders (≥1 h), statistically significant improvements in depression scores were observed with safinamide 50 and 100 mg compared with placebo (*p* = 0.0011 and *p* = 0.0025, respectively). Conversely, in the ON-time low/non-responders (<1 h), no improvement in depression scores was observed.

In the subgroup with depression at baseline (a score of >0 on UPDRS Part I item 3), a significant improvement was observed only with safinamide 100 mg (*p* = 0.0167). A significant improvement in depression was also observed in the subgroup with pain during OFF-time at baseline, with both safinamide 50- and 100-mg (*p* = 0.0099 and *p* = 0.0161, respectively) (Supplementary Table 3).

At baseline, the UPDRS Part I item 3 score was higher in female patients than in male patients. A statistically significant improvement in depression was observed in the female subgroup with both safinamide 50 and 100 mg, compared with the placebo group (*p* = 0.0083 and *p* = 0.0177, respectively). In the male subgroup, there was no significant improvement in depression, although there was a numerical dose-related improvement.

A statistically significant improvement in depression score was observed with safinamide 50- and 100-mg compared with placebo in patients receiving concomitant dopamine agonists at baseline (*p* = 0.0137 and *p* = 0.0070, respectively). In groups either receiving and not receiving dopamine agonists, point estimates of the difference between the safinamide groups and the placebo group were <0, indicating the same tendency toward improvement observed in the FAS.

### Efficacy of Safinamide on Improving Apathy (Subgroup Analyses)

**Figure 2:**
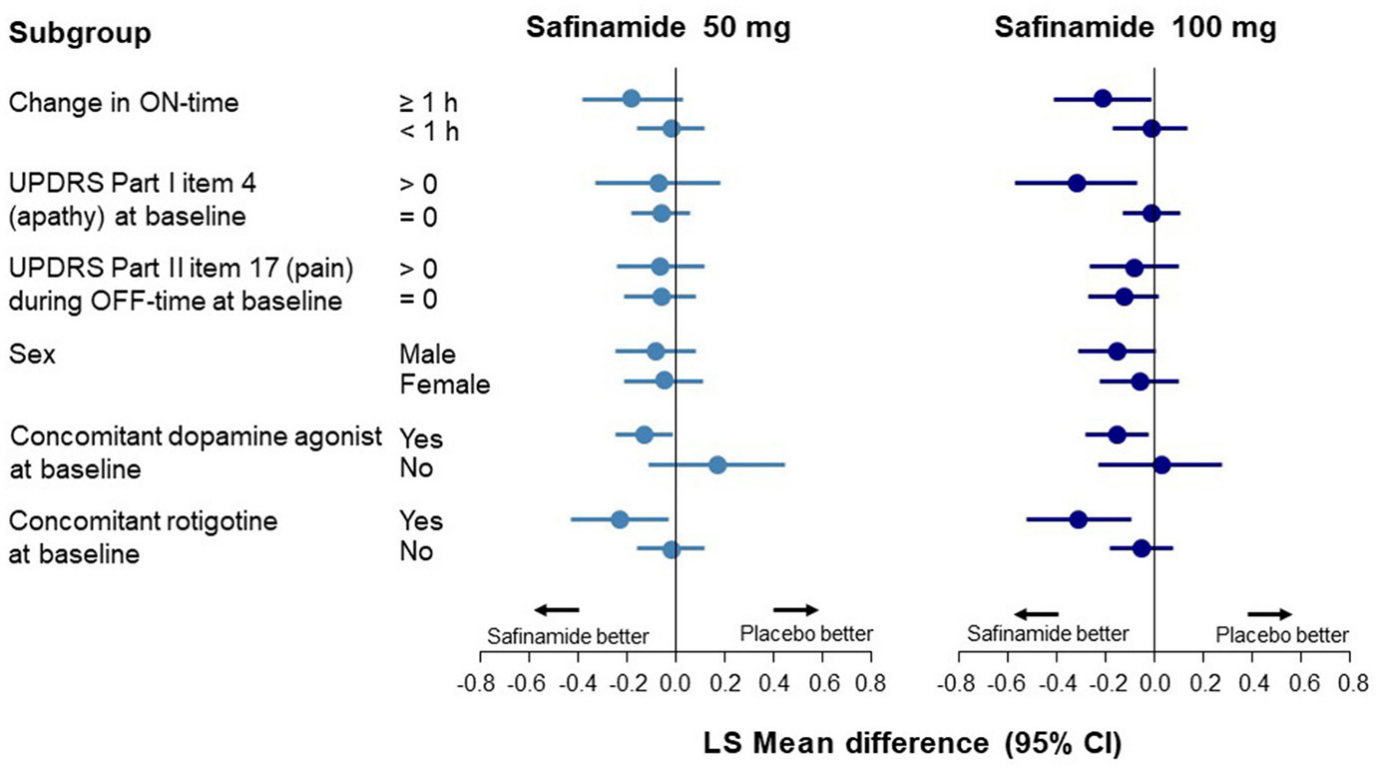
Forest plot of change from baseline to Week 24 in the UPDRS Part I item 4 score according to subgroup. CI, confidence interval; UPDRS, Unified Parkinson's Disease Rating Scale.

Data corresponding to changes from baseline to Week 24 in UPDRS Part I item 4 scores are shown in Figure 2 and Supplementary Table 4. An improvement in apathy was observed only in the ON-time high responders; this improvement was statistically significant with safinamide 100 mg (*p* = 0.0382). In the subgroup with apathy at baseline (a score of >0 on UPDRS Part I item 4), a significant improvement was observed only in the 100-mg group (*p* = 0.0127). No improvement was observed in either safinamide dose group in patients with pain at baseline, which was in contrast to the results for depression. A significant improvement in apathy was observed in both the safinamide 50- and 100-mg groups for patients using concomitant dopamine agonists (safinamide 50 mg, *p* = 0.0403; 100 mg, *p* = 0.0201).

### Change in ON-Time in Specific Subgroups

The potential associations observed between the effect of safinamide on neuropsychiatric symptoms and change in ON-time or concomitant medications were further evaluated by analyzing changes in ON-time according to the concomitant use of dopamine agonists at baseline (Supplementary Table 5). ON-time notably improved in the subgroup using concomitant dopamine agonists compared with the subgroup without these drugs.

## Discussion

In this *post-hoc* analysis of a placebo-controlled phase 2/3 study in Japanese patients with PD (33), we investigated the impact of adjunct safinamide 50 and 100 mg on depression and apathy, two neuropsychiatric symptoms which are known to significantly affect the QOL of patients with PD (10, 14, 34). The study results demonstrated the potential benefit of safinamide in alleviating these neuropsychiatric symptoms, particularly depression.

In the FAS analysis, the UPDRS Part I item 3 (depression) score was significantly improved in both safinamide dose groups compared with the placebo group. This outcome is consistent with data reported from a prior *post-hoc* analysis of the global phase 3 studies (32). Conversely, PDQ-39 emotional well-being scores were numerically, but not significantly, improved by safinamide in a dose-related manner. One of the possible reason for why PDQ-39 emotional well-being did not reach statistical significance might be because of small number of subjects and high variability of scores at baseline in the Japanese study compared to the global phase 3 study (35). However, the change from baseline was smaller than 0 in both safinamide dose groups, which was consistent with the improvement in neuropsychiatric symptoms suggested by the change in the UPDRS Part I item 3 scores. In the prior *post-hoc* analysis of the global phase 3 studies, both the subjective PDQ-39 emotional well-being scores and the objective GRID-HAMD scores significantly decreased in patients receiving safinamide 100 mg, indicating that safinamide improved depression (32). The results of the present analysis supported the effect of safinamide on objective depressive symptoms in the Japanese population.

It has been hypothesized previously that depression in PD could be related to disease factors, including the severity and fluctuating variability of motor symptoms (15), non-motor symptoms (e.g., pain) (36), and sleep disorders (37), as well as patient background factors, such as disease duration (38) and sex (39). Many of these characteristics are consistent with the demography of patients with depressive symptoms in the current analysis. In this analysis, we conducted subgroup analyses to identify patient populations expected to obtain neuropsychiatric benefit from safinamide treatment. Notable improvements in depression were observed in ON-time high responders, patients with pain during OFF-time at baseline, and female patients. It is known that neuropsychiatric symptoms in PD can be exacerbated during the OFF phase (23–25), indicating that a reduction in OFF-time can improve symptoms. The data from this *post-hoc* analysis confirmed an improvement in ON-time (and thus, a decrease in OFF-time) contributes to improved depressive symptoms. Furthermore, in the subgroup analysis, patients using concomitant dopamine agonists at baseline also had a significant improvement in depression. There was a notable improvement of ON-time in these patients, which may have contributed to the improvements in depression. However, the number of patients not using concomitant dopamine agonists at baseline was small, which may have resulted in insufficient statistical power to fully evaluate the data.

Pain is a factor that has been previously linked to depression in PD (17), so pain relief may lead to improved depression. In a previously published *post-hoc* analysis of global safinamide studies (40), an improvement in PDQ-39 items 37 and 39 was observed following treatment with safinamide 100 mg for 24 weeks. In the current analysis, an improvement in depression was observed in the subgroup with UPDRS item 17 (OFF) scores of >0 at baseline. These data suggest that the improvement in depression produced by safinamide may be partially due to alleviation of pain. Sex is another well-characterized factor affecting depression in PD (39, 41, 42), and prior studies have reported that depression scores are often higher in females than in males (39). Indeed, in this analysis, the baseline UPDRS Part I item 3 score was found to be higher in females than in males. Safinamide improved depression in female patients, even at the lower (50-mg) dose, and we consider that this improvement may be linked to the higher baseline depression scores.

It is thought that around half of patients with PD who develop depression do so as a direct result of the pathophysiological mechanisms underlying PD (43). In patients with PD, neurological deficits in dopamine production have been observed not only within the substantia nigra but also in the ventral tegmental area (VTA) of the brain (44, 45). It is considered that the decrease of dopamine output from the VTA to the ventral striatum may contribute to dysfunction in the orbitofrontal cortex and anterior cingulate cortex, and ultimately result in depression (46). In this study, safinamide provided improvements in depression even at the lower (50-mg) dose, suggesting that its dopaminergic effects contributed to the improvement of depression. Some patients can obtain improvements in depression and the motor symptoms of PD following treatment with supplemental dopamine therapy such as levodopa and dopamine agonists; however, other patients may require additional therapies to improve neuropsychiatric symptoms (26). Safinamide can act as a reversible MAO-B inhibitor and also as an inhibitor of glutamate release via voltage-gated sodium-channels (31). Since the 50-mg dose of safinamide can almost inhibit MAO-B (47), the efficacy of the 100-mg dose in the subgroup with baseline depression may be explained instead by the non-dopaminergic action of safinamide. However, this speculation remains to be confirmed in future studies.

In the present analysis, a significant improvement in apathy following safinamide treatment was observed in the subgroup with apathy at baseline. The major pathological mechanism underlying apathy is a dopaminergic dysfunction in the mesocorticolimbic pathway (48), so it is generally considered that dopamine agonists are an effective treatment for apathy in PD (49). Consistent with this reasoning, because safinamide act as MAO-B inhibitor, safinamide's dopaminergic action may contribute to its improvement of apathy. In addition, in the subgroup analyses, safinamide notably improved apathy in the ON-time high responders. Patients with concomitant baseline dopamine agonist also had a notable change in ON-time with safinamide treatment. Like depression, apathy is a non-motor symptom that can show exacerbation during the OFF phase (50). Considering the results obtained in the subgroup analyses, the improvements in daily motor fluctuations produced by safinamide may contribute to apathy improvement. It should, however, be noted that due to the small number of patients without concomitant dopamine agonists at baseline, the statistical power to fully analyze these data was insufficient. Notably, apathy in PD is also related to executive function (51), and in a recent observational study, a 100-mg dose of safinamide was shown to improve executive function (52). Thus, the improvement of apathy by safinamide may also involve amelioration of cognitive dysfunction.

Several study limitations should be considered when interpreting the data from our analyses. First, this was a *post-hoc* analysis, and the endpoints and calculations were not predefined before the initiation of clinical procedures. The small number of patients may have confounded the statistical power in some subgroups, and the data are also limited by the lack of adjustment for differences in baseline values among the subgroups. The effects of safinamide on neuropsychiatric symptoms might be confounded in the present study because of the small proportion of patients who had mood disturbances at baseline and had milder depressive symptoms than other studies (53, 54), meaning further investigations are necessary to evaluate the effect of safinamide on neuropsychiatric symptoms in patients with PD who have moderate or severe depressive symptoms in the real world. Second, in this study, depression was assessed with a scale specific to depressive symptoms in PD rather than a general scale for depression, potentially reducing the ability to correlate outcomes across different studies. Therefore, further studies are needed using recommended assessment scale for depression such as Beck Depression Inventory and Hospital Anxiety and Depression Scale (55). Finally, it is also clear that neuropsychiatric symptoms are closely related to various other PD symptoms. We cannot eliminate the possibility that safinamide acts only indirectly on depression and apathy via improvement of those other symptoms, rather than having a direct effect.

In conclusion, the results from this *post-hoc* analysis of the Japanese phase 2/3 study suggest that the impact of safinamide on depression and apathy is related to its efficacy in improving motor fluctuations. Safinamide improved depression in the overall patient population and across multiple subgroups and improved apathy in patients with apathy at baseline. We consider that safinamide may provide particular benefits for patients with PD who have mild depression and/or apathy, pain during the OFF phase and female.

## Data Availability Statement

The raw data supporting the conclusions of this article will be made available by the authors, without undue reservation.

## Ethics Statement

The studies involving human participants were reviewed and approved by all appropriate independent ethics committees and the Japanese regulatory authority (the full list is available as a Supplementary File). The patients/participants provided their written informed consent to participate in this study.

## Author Contributions

NH, YK, MK, TI, IS, YT, and MN conceived and designed the study, interpreted the data, reviewed the manuscript drafts, and provided important intellectual input. IS conducted the statistical analysis. YK prepared the first draft of the manuscript. All authors approved the final manuscript.

## Funding

This work was sponsored by Eisai Co., Ltd. The clinical trial was sponsored by Meiji Seika Pharma Co., Ltd. with no further involvement in the study.

## Conflict of Interest

The authors declare that this study received funding from Eisai Co., Ltd. The funder had the following involvement in the study: study design, data collection, analysis, interpretation of data, and in the decision to submit the article for publication.

## Publisher's Note

All claims expressed in this article are solely those of the authors and do not necessarily represent those of their affiliated organizations, or those of the publisher, the editors and the reviewers. Any product that may be evaluated in this article, or claim that may be made by its manufacturer, is not guaranteed or endorsed by the publisher.
