# Supplementary material for: The Effects of Safinamide Adjunct Therapy on Depression and Apathy in Patients With Parkinson's Disease: Post-hoc Analysis of a Japanese Phase 2/3 Study
Source: Front Neurol. 2022 Feb 7;12:752632. doi: 10.3389/fneur.2021.752632 (PMC8869178; doi:10.3389/fneur.2021.752632)
Supplement: Supplementary file 1 [file Table_1.docx]

**Supplementary Table 1 |** Demographic and clinical characteristics of patients.

|  | Placebo | Safinamide 50 mg | Safinamide 100 mg |
| --- | --- | --- | --- |
| *N* | 136 | 131 | 128 |
| Age (years), mean ± SD | 68.64 ± 7.66 | 67.18 ± 9.04 | 68.36 ± 9.04 |
| Sex (male), *n* (%) | 57 (41.9) | 58 (44.3) | 63 (49.2) |
| Duration of PD (years), mean ± SD | 7.97 ± 5.10 | 8.23 ± 4.16 | 8.55 ± 5.37 |
| ≥10 years, *n* (%) | 38 (27.9) | 45 (34.4) | 38 (29.7) |
| Duration of treatment with levodopa (years), mean ± SD | 5.55 ± 4.31 | 6.04 ± 4.06 | 6.41 ± 5.04 |
| Duration of wearing off phenomenon (years), mean ± SD | 2.69 ± 2.79 | 2.82 ± 2.54 | 3.09 ± 3.13 |
| Modified H&Y stage, mean ± SD |  |  |  |
| ON phase | 2.33 ± 0.59 | 2.34 ± 0.62 | 2.41 ± 0.62 |
| OFF phase | 3.14 ± 0.63 | 3.17 ± 0.67 | 3.25 ± 0.61 |
| Mean daily ON-time without troublesome dyskinesia (hours), mean ± SD | 10.41 ± 2.69 | 9.87 ± 2.92 | 9.52 ± 3.01 |
| Mean daily OFF-time (hours), mean ± SD | 5.66 ± 2.56 | 6.22 ± 2.81 | 6.49 ± 3.17 |
| Dose of levodopa at baseline (mg), mean ± SD | 420.04 ± 123.90 | 445.61 ± 156.66 | 446.48 ± 153.49 |
| UPDRS scores, mean ± SD |  |  |  |
| Part I | 1.10 ± 1.45 | 1.25 ± 1.70 | 1.34 ± 1.66 |
| Part II (ON phase) | 5.25 ± 4.39 | 5.63 ± 4.89 | 6.30 ± 5.02 |
| Part II (OFF phase) | 13.12 ± 7.23 | 14.51 ± 6.59 | 15.00 ± 7.75 |
| Part III (ON phase) | 21.78 ± 10.35 | 21.18 ± 11.18 | 23.13 ± 12.40 |
| Part IV | 4.24 ± 1.96 | 4.51 ± 1.85 | 4.76 ± 2.22 |
| Item 3 | 0.31 ± 0.55 | 0.29 ± 0.56 | 0.33 ± 0.68 |
| Item 4 | 0.39 ± 0.66 | 0.40 ± 0.70 | 0.40 ± 0.66 |
| PDQ-39 summary index score, mean ± SD | 21.59 ± 12.50 | 24.94 ± 15.39 | 26.62 ± 14.79 |
| Emotional well-being domain score | 23.28 ± 17.69 | 29.26 ± 21.27 | 29.20 ± 22.35 |

H&Y, Hoehn & Yahr; PD, Parkinson’s disease; PDQ-39, 39-item Parkinson's Disease Questionnaire; SD, standard deviation; UPDRS, Unified Parkinson's Disease Rating Scale.

**Supplementary Table 2 |** Baseline demographic and clinical characteristics according to baseline UPDRS Part I item 3 score (> 0 or 0) and between-group statistical comparisons.

|  | **UPDRS Part I item 3** | |  |
| --- | --- | --- | --- |
|  | **Score > 0** | **Score 0** | ***p* value** |
| *N* | 98 | 297 | - |
| Age (years), mean ± SD | 69.60 ± 8.40 | 67.56 ± 8.61 | 0.0394^a^ |
| Sex (male), *n* (%) | 32 (32.7) | 146 (49.2) | 0.0049^b^ |
| Duration of PD (years), mean ± SD | 9.16 ± 4.44 | 7.94 ± 5.01 | 0.0242^a^ |
| ≥10 years, *n* (%) | 40 (40.8) | 81 (27.3) | 0.0160^b^ |
| Duration of treatment with levodopa (years), mean ± SD | 6.78 ± 4.43 | 5.73 ± 4.48 | 0.0434^a^ |
| Duration of wearing off phenomenon (years), mean ± SD | 2.84 ± 2.39 | 2.87 ± 2.96 | 0.9167^a^ |
| Modified H&Y stage, mean ± SD |  |  |  |
| ON phase | 2.36 ± 0.53 | 2.36 ± 0.63 | 0.9763^a^ |
| OFF phase | 3.38 ± 0.64 | 3.12 ± 0.62 | 0.0007^a^ |
| Mean daily ON-time without troublesome dyskinesia (hours), mean ± SD | 9.21 ± 2.98 | 10.18 ± 2.83 | 0.0053^a^ |
| Mean daily OFF-time (hours), mean ± SD | 6.66 ± 2.92 | 5.93 ± 2.83 | 0.0324^a^ |
| Dose of levodopa at baseline (mg), mean ± SD | 465.31 ± 168.48 | 427.78 ± 135.80 | 0.0473^a^ |
| UPDRS scores, mean ± SD |  |  |  |
| Part I | 3.07 ± 1.63 | 0.62 ± 1.03 | <0.0001^a^ |
| Part II (ON phase) | 6.91 ± 4.15 | 5.32 ± 4.91 | 0.0020^a^ |
| Part II (OFF phase) | 18.81 ± 7.05 | 12.67 ± 6.62 | <0.0001^a^ |
| Part III (ON phase) | 25.66 ± 12.19 | 20.81 ± 10.7 | 0.0006^a^ |
| Part IV | 4.59 ± 2.02 | 4.47 ± 2.02 | 0.5995^a^ |
| Item 17 (ON phase) | 0.40 ± 0.71 | 0.30 ± 0.65 | 0.2453^a^ |
| Item 17 (OFF phase) | 0.98 ± 1.16 | 0.72 ± 1.02 | 0.0527^a^ |
| PDQ-39 summary index score, mean ± SD | 32.04 ± 13.90 | 21.79 ± 13.62 | <0.0001^a^ |
| Emotional well-being domain score, mean ± SD | 40.69 ± 21.80 | 22.73 ± 18.17 | <0.0001^a^ |

H&Y, Hoehn & Yahr; PD, Parkinson’s disease; PDQ-39, 39-item Parkinson's Disease Questionnaire; SD, standard deviation; UPDRS, Unified Parkinson's Disease Rating Scale. ^a^ Welch’s t-test; ^b^ Fisher’s exact test.

**Supplementary Table 3 |** Baseline values and change from baseline to Week 24 in the UPDRS Part I item 3 score by subgroup.

|  | **Placebo** | **Safinamide  50 mg** | **Safinamide  100 mg** | **Placebo** | **Safinamide  50 mg** | **Safinamide  100 mg** |
| --- | --- | --- | --- | --- | --- | --- |
| **Change from baseline at Week 24 in ON-time without troublesome dyskinesia** | **High responder (≥ 1 hour)** | | | **Low/non-responder (< 1 hour)** | | |
| *N* | 35 | 63 | 79 | 101 | 68 | 49 |
| Baseline, mean ± SD | 0.40 ± 0.65 | 0.24 ± 0.50 | 0.38 ± 0.77 | 0.28 ± 0.51 | 0.34 ± 0.61 | 0.24 ± 0.48 |
| Change from baseline at Week 24 (LOCF), LS mean ± SE | 0.10 ± 0.06 | −0.17 ± 0.05 | −0.14 ± 0.04 | 0.08 ± 0.04 | 0.01 ± 0.05 | 0.01 ± 0.06 |
| LS mean difference vs placebo [95% CI] | — | −0.26 [−0.42, 0.11] | −0.23 [−0.38, −0.08] | — | −0.07 [−0.20, 0.07] | −0.07 [−0.21, 0.08] |
| *p* value vs placebo | — | 0.0011 | 0.0025 | — | 0.3283 | 0.3801 |
| **UPDRS Part I item 3 (depression) at baseline** |  | **Score > 0** |  |  | **Score 0** |  |
| *N* | 36 | 31 | 31 | 100 | 100 | 97 |
| Baseline, mean ± SD | 1.17 ± 0.38 | 1.23 ± 0.43 | 1.35 ± 0.71 | 0.00 ± 0.00 | 0.00 ± 0.00 | 0.00 ± 0.00 |
| Change from baseline at Week 24 (LOCF), LS mean ± SE | −0.18 ± 0.10 | −0.46 ± 0.11 | −0.56 ± 0.11 | 0.15 ± 0.03 | 0.07 ± 0.03 | 0.07 ± 0.03 |
| LS mean difference vs placebo [95% CI] | — | −0.28 [−0.59, 0.02] | −0.38 [−0.68, −0.07] | — | −0.08 [−0.17, 0.01] | −0.08 [−0.17, 0.01] |
| *p* value vs placebo | — | 0.0680 | 0.0167 | — | 0.0863 | 0.0976 |
| **UPDRS Part II item 17 (pain) during OFF-time at baseline** |  | **Score > 0** |  |  | **Score 0** |  |
| *N* | 59 | 61 | 56 | 77 | 70 | 72 |
| Baseline, mean ± SD | 0.27 ± 0.45 | 0.31 ± 0.53 | 0.43 ± 0.76 | 0.34 ± 0.62 | 0.27 ± 0.59 | 0.25 ± 0.60 |
| Change from baseline at Week 24 (LOCF), LS mean ± SE | 0.12 ± 0.06 | −0.11 ± 0.06 | −0.10 ± 0.07 | 0.03 ± 0.04 | −0.02 ± 0.04 | −0.07 ± 0.04 |
| LS mean difference vs placebo [95% CI] | — | −0.23 [−0.41, -0.06] | −0.22 [−0.40, −0.04] | — | −0.05 [−0.17, 0.07] | −0.10 [−0.22, 0.02] |
| *p* value vs placebo | — | 0.0099 | 0.0161 | — | 0.3808 | 0.0878 |
| **Sex** |  | **Male** |  |  | **Female** |  |
| *N* | 57 | 58 | 63 | 79 | 73 | 65 |
| Baseline, mean ± SD | 0.25 ± 0.58 | 0.22 ± 0.53 | 0.29 ± 0.73 | 0.35 ± 0.53 | 0.34 ± 0.58 | 0.37 ± 0.63 |
| Change from baseline at Week 24 (LOCF), LS mean ± SE | 0.03 ± 0.05 | −0.03 ± 0.05 | −0.09 ± 0.05 | 0.10 ± 0.05 | −0.09 ± 0.05 | −0.07 ± 0.05 |
| LS mean difference vs placebo [95% CI] | — | −0.06 [−0.21, 0.08] | −0.12 [−0.27, 0.02] | — | −0.19  [−0.33, −0.05] | −0.17 [−0.32, −0.03] |
| *p* value vs placebo | — | 0.3916 | 0.0890 | — | 0.0083 | 0.0177 |
| **Concomitant dopamine agonist at baseline** |  | **Yes** |  |  | **No** |  |
| *N* | 97 | 109 | 96 | 39 | 22 | 32 |
| Baseline, mean ± SD | 0.33 ± 0.57 | 0.28 ± 0.56 | 0.30 ± 0.62 | 0.26 ± 0.50 | 0.32 ± 0.57 | 0.41 ± 0.84 |
| Change from baseline at Week 24 (LOCF), LS mean ± SE | 0.10 ± 0.04 | −0.04 ± 0.04 | −0.06 ± 0.04 | −0.01 ± 0.07 | −0.14 ± 0.09 | −0.15 ± 0.07 |
| LS mean difference vs placebo [95% CI] | — | −0.15 [−0.26, −0.03] | −0.17 [−0.29, −0.05] | — | −0.13 [−0.35, 0.09] | −0.14 [−0.33, 0.06] |
| *p* value vs placebo | — | 0.0137 | 0.0070 | — | 0.2348 | 0.1633 |

CI, confidence intervals; LOCF, last observation carried forward; LS, least squares; SD, standard deviation; SE, standard error; UPDRS, Unified Parkinson’s Disease Rating Scale.

**Supplementary Table 4.** Baseline values and change from baseline to Week 24 in the UPDRS Part I item 4 score by subgroup.

|  | **Placebo** | **Safinamide  50 mg** | **Safinamide  100 mg** | **Placebo** | **Safinamide  50 mg** | **Safinamide  100 mg** |
| --- | --- | --- | --- | --- | --- | --- |
| **Change from baseline at Week 24 in ON-time without troublesome dyskinesia** | **High responder (≥ 1 hour)** | | | **Low/non-responder (< 1 hour)** | | |
| *N* | 35 | 63 | 79 | 101 | 68 | 49 |
| Baseline, mean ± SD | 0.43 ± 0.70 | 0.29 ± 0.58 | 0.44 ± 0.69 | 0.38 ± 0.65 | 0.50 ± 0.78 | 0.33 ± 0.59 |
| Change from baseline at Week 24 (LOCF), LS mean ± SE | 0.13 ± 0.08 | −0.04 ± 0.06 | −0.08 ± 0.06 | 0.03 ± 0.04 | 0.01 ± 0.05 | 0.02 ± 0.06 |
| LS mean difference vs placebo [95% CI] | — | −0.18 [−0.38, 0.03] | −0.21 [−0.41, −0.01] | — | −0.02 [−0.16, 0.12] | −0.01 [−0.17, 0.14] |
| *p* value vs placebo | — | 0.0904 | 0.0382 | — | 0.8195 | 0.8573 |
| **UPDRS Part I item 4 (apathy) at baseline** |  | **Score > 0** |  |  | **Score 0** |  |
| *N* | 40 | 36 | 39 | 96 | 95 | 89 |
| Baseline, mean ± SD | 1.33 ± 0.47 | 1.44 ± 0.50 | 1.31 ± 0.47 | 0.00 ± 0.00 | 0.00 ± 0.00 | 0.00 ± 0.00 |
| Change from baseline at Week 24 (LOCF), LS mean ± SE | −0.23 ± 0.09 | −0.31 ± 0.09 | −0.55 ± 0.09 | 0.18 ± 0.04 | 0.12 ± 0.04 | 0.17 ± 0.05 |
| LS mean difference vs placebo [95% CI] | — | −0.07 [−0.33, 0.18] | −0.32 [−0.57, −0.07] | — | −0.06 [−0.18, 0.06] | −0.01 [−0.13, 0.11] |
| *p* value vs placebo | — | 0.5629 | 0.0127 | — | 0.3206 | 0.8916 |
| **UPDRS Part II item 17 (pain) during OFF-time at baseline** |  | **Score > 0** |  |  | **Score 0** |  |
| *N* | 59 | 61 | 56 | 77 | 70 | 72 |
| Baseline, mean ± SD | 0.46 ± 0.68 | 0.43 ± 0.72 | 0.48 ± 0.71 | 0.34 ± 0.64 | 0.37 ± 0.68 | 0.33 ± 0.61 |
| Change from baseline at Week 24 (LOCF), LS mean ± SE | 0.03 ± 0.06 | −0.02 ± 0.06 | −0.05 ± 0.07 | 0.07 ± 0.05 | 0.01 ± 0.05 | −0.05 ± 0.05 |
| LS mean difference vs placebo [95% CI] | — | −0.06 [−0.24, 0.12] | −0.08 [−0.26, 0.10] | — | −0.06 [−0.21, 0.08] | −0.12 [−0.27, 0.02] |
| *p* value vs placebo | — | 0.5128 | 0.3874 | — | 0.3953 | 0.1006 |
| **Sex** |  | **Male** |  |  | **Female** |  |
| *N* | 57 | 58 | 63 | 79 | 73 | 65 |
| Baseline, mean ± SD | 0.30 ± 0.60 | 0.31 ± 0.60 | 0.35 ± 0.63 | 0.46 ± 0.69 | 0.47 ± 0.77 | 0.45 ± 0.69 |
| Change from baseline at Week 24 (LOCF), LS mean ± SE | 0.10 ± 0.06 | 0.01 ± 0.06 | −0.05 ± 0.06 | 0.03 ± 0.06 | −0.02 ± 0.06 | −0.03 ± 0.06 |
| LS mean difference vs placebo [95% CI] | — | −0.08 [−0.25, 0.08] | −0.15 [−0.31, 0.01] | — | −0.05  [−0.21, 0.11] | −0.06 [−0.22, 0.10] |
| *p* value vs placebo | — | 0.3050 | 0.0591 | — | 0.5474 | 0.4743 |
| **Concomitant dopamine agonist at baseline** |  | **Yes** |  |  | **No** |  |
| *N* | 97 | 109 | 96 | 39 | 22 | 32 |
| Baseline, mean ± SD | 0.41 ± 0.67 | 0.39 ± 0.69 | 0.40 ± 0.67 | 0.33 ± 0.62 | 0.45 ± 0.74 | 0.41 ± 0.61 |
| Change from baseline at Week 24 (LOCF), LS mean ± SE | 0.10 ± 0.05 | −0.03 ± 0.04 | −0.05 ± 0.05 | −0.05 ± 0.08 | 0.12 ± 0.11 | −0.02 ± 0.09 |
| LS mean difference vs placebo [95% CI] | — | −0.13 [−0.25, −0.01] | −0.15 [−0.28, −0.02] | — | 0.17 [−0.11, 0.45] | 0.03 [−0.23, 0.28] |
| *p* value vs placebo | — | 0.0403 | 0.0211 | — | 0.2366 | 0.8400 |

CI, confidence intervals; LOCF, last observation carried forward; LS, least squares; SD, standard deviation; SE, standard error; UPDRS, Unified Parkinson’s Disease Rating Scale.

**Supplementary Table 5 |** Baseline values and change from baseline to Week 24 in ON-time by selected subgroup.

|  | **Placebo** | **Safinamide  50 mg** | **Safinamide  100 mg** | **Placebo** | **Safinamide  50 mg** | **Safinamide  100 mg** |
| --- | --- | --- | --- | --- | --- | --- |
| **Concomitant dopamine agonist at baseline** |  | **Yes** |  |  | **No** |  |
| *N* | 97 | 109 | 96 | 39 | 22 | 32 |
| Baseline, mean ± SD | 10.62 ± 2.77 | 9.89 ± 2.75 | 9.56 ± 2.76 | 9.89 ± 2.44 | 9.80 ± 3.74 | 9.38 ± 3.71 |
| Change from baseline at Week 24 (LOCF), LS mean ± SE | −0.39 ± 0.29 | 1.25 ± 0.27 | 1.48 ± 0.29 | 0.61 ± 0.49 | 1.04 ± 0.65 | 1.45 ± 0.54 |
| LS mean difference vs placebo [95% CI] | — | 1.65 [0.87, 2.43] | 1.87 [1.06, 2.68] | — | 0.44 [−1.19, 2.06] | 0.84 [−0.62, 2.30] |
| *p* value vs placebo | — | <0.0001 | <0.0001 | — | 0.5951 | 0.2564 |

CI, confidence intervals; LOCF, last observation carried forward; LS, least squares; SD, standard deviation; SE, standard error.
