# Supplementary material for: The Effects of Safinamide Adjunct Therapy on Depression and Apathy in Patients With Parkinson's Disease: Post-hoc Analysis of a Japanese Phase 2/3 Study
Source: Front Neurol. 2022 Feb 7;12:752632. doi: 10.3389/fneur.2021.752632 (PMC8869178; doi:10.3389/fneur.2021.752632)
Supplement: Supplementary file 2 [file Data_Sheet_1.docx]

The list of the ethics committees.

| Aizawa Hospital |
| --- |
| Aomori Prefectural Central Hospital |
| Ebara Hospital |
| Ehime University Hospital |
| Federation of National Public Service Personnel Mutual Aid Associations Tachikawa Hospital |
| Fukuoka University Hospital |
| Haradoi Hospital |
| Himeji Central Hospital |
| Hospital of the University of Occupational and Environmental Health |
| Ina Central Hospital |
| Irahara Clinic |
| Iwamizawa Neurology Clinic |
| Iwate Medical University |
| Japan Organization of Occupational Health and Safety Chugoku Rosai Hospital |
| Japan Organization of Occupational Health and Safety Tokyo Rosai Hospital |
| Japanese Red Cross Society Nagano Red Cross Hospital |
| Juntendo University Hospital |
| Juntendo University Nerima Hospital |
| Juntendo University Shizuoka Hospital |
| Juntendo University Urayasu Hospital |
| Junwa Rehabilitation Foundation Junwakai Memorial Hospital |
| Kanto Central Hospital of the Mutual Aid Association of Public School Teachers |
| Kitamachi Clinic |
| Local Independent Administrative Agency Osaka Prefectural Hospital Organization Osaka General Medical Center |
| Minoh City Hospital |
| Nagoya Ishikai |
| Nara City Hospital |
| National Center of Neurology and Psychiatry |
| National Hospital Organization Asahikawa Medical Center |
| National Hospital Organization Chiba-East-Hospital |
| National Hospital Organization Higashinagoya National Hospital |
| National Hospital Organization Hokkaido Medical Center |
| National Hospital Organization Kumamoto Minami National Hospital |
| National Hospital Organization Maizuru Medical Center |
| National Hospital Organization Minami-Kyoto Hospital |
| National Hospital Organization Nagoya Medical Center |
| National Hospital Organization Nishiniigata Chuo Hospital |
| National Hospital Organization Omuta Hospital |
| National Hospital Organization Sagamihara National Hospital |
| National Hospital Organization Saigata Medical Center |
| National Hospital Organization Sendai Medical Center |
| National Hospital Organization Sendai-Nishitaga Hospital |
| National Hospital Organization Shizuoka Institute of Epilepsy and Neurological Disorders |
| National Hospital Organization Tokushima National Hospital |
| National Hospital Organization Toneyama National Hospital |
| National Hospital Organization Utano Hospital |
| Nitobe Memorial Nakano General Hospital |
| Okayama Kyokuto Hospital |
| Osaka University Hospital |
| Review Board of Human Rights and Ethics for Clinical Studies |
| Seirei Hamamatsu General Hospital |
| Takamatsu Neurology Clinic |
| Tatsuoka Neurology Clinic |
| Teine Keijinkai Hospital |
| Tohoku University Hospital |
| Tokushukai Group |
| Tokyo Metropolitan Geriatric Hospital |
| Tokyo Metropolitan Neurological Hospital |
| Tominaga Hospital |
| Tsuchiura Kyodo General Hospital |
| University of Tsukuba Hospital |
| Wakayama Medical University Hospital |
| Yokohama Minoru Clinic |
